# On the Physiological Antagonism between Medicines, and between Remedies and Diseases

**Published:** 1880-11

**Authors:** Roberts Bartholow

**Affiliations:** Professor of Materia Medica and Therapeutics in the Jefferson Medical College, Philadelphia


### ON THE PHYSIOLOGICAL ANTAGONISM BETWEEN MEDICINES, AND BETWEEN REMEDIES AND DISEASES.

BY ROBERTS BARTHOLOW, M.D.,
Professor of Materia Medica and Therapeutics in the Jefferson Medical College, Philadelphia.

We extract the following valuable lecture on this important subject from the *New York Medical Record.*

*General Introduction—The Physiological Antagonism Between Opium and Belladonna.*—Mr. President and Gentlemen—In this country private benificence, although vast in its generous outlays, is seldom devoted to purely medical objects. It, therefore, behooves us to honor the memory of the founder of the Cartwright lectureship. In this gist, so rare and admirable in its provisions, the medical profession is offered an opportunity for honorable distinction; while it is replete with benefits not less to the founder than to the profession. By this means his benefactions are especially celebrated annually, and he is enrolled for all future time among the philanthropists who really benefit their fellow-men.

I cannot be insensible to the very high honor of having been selected to deliver the first course of these lectures; but I do not conceal from myself the difficulties and obligations of the position. In the Gulstanian, Lumlian, and other lecture courses which have done such good work in England, it is customary to expect that new facts, new truths, and new discoveries will be set forth; and brought into comparison with such admirable efforts, I can but feel how humble are the attempts to which I am to ask your attention. After careful consideration, however, I have selected a topic which has a strong interest at the present day, and to which I have been able of late to add some few new facts.

My subject is the Physiological Antagonism between Medicines, and between Remedies and Diseases; and this antagonism or opposition of action may exist throughout the whole range of the relations of such agents, or it may be limited to a few points only. There are, however, few, if any, in which the antagonism is universal. The popular view of the doctrine of antagonisms in the profession is an opposition of action of poisonous medicinal agents, by which the effects produced by one are counteracted and rendered void by those of the other. A dose of one may thus be completely antagonized by a dose of the other. This conception of physiological antagonism is exaggerated; for such a perfect antagonism is as rare as exact similitude. It has been proposed by some to substitute the word antidotism for antagonism; but this implies a chemical, rather than a physiological action. Physiological antagonism means simply a balance of opposing agents.

Some such conception as our modern doctrine of physiological antagonism has existed from the remotest times, as is evident from some of the aphorisms of Hippocrates. Thus he speaks of repletion being counteracted by depletion, and says that diseases are cured by their contraries. In another aphorism he says that some diseases are cured by contraries, and some by remedies. On this was founded the well-known criticism of Carus in his controversy with Hahnemann: "Whatever is new in homœopathy is not true, and whatever is true in it is not new." The doctrine of contraries was held by Theophrastus, and many others who succeeded Hippocrates; but it was opposed by some of the most distinguished of the Alexandrian school Galen, however, was essentially a trimmer, and he "claimed to create a science of medicine in which every practical precept was deduced from some pre-established principle." But the only rational expedient in medicine applicable at that time was the doctrine of contraries, which controlled the practice of the whole profession until the sixteenth century. Fernel, the teacher of Versalius wrote an elaborate treatise in which he contended that every disease must be controlled by contraries. Some claimed, he stated, that certain diseases were cured by similars; but this *modus operandi* was only apparent, and not real, since remedies, which appeared to act in this way, in fact destroyed the disease by removing its cause.

This ingenious attempt of Fernel, however, could not prevent the final overthow of the doctrine of contraries, and when Paracelsus burned the works of Galen and Avicenna, it vanished away in their smoke. After the discovery of the circulation of the blood many new theories were advanced, and the systems of Stahi, Brown, Rasori, and others necessarily attracted the attention of the profession Then arose that eccentric and mystical enthusiast, Hahnemann, imbued with the fanciful ideas of Mesmer. Before the period of his senility, which was especially characterized by his spiritualistic tendencies, the law of similars propounded by him was merely a statement of the views of Hippocrates, to which allusion has already been made. Both the laws of contraries and the law of similars were stated by Hippocretes, as we have seen. Hahnemann claimed that the disturbance in the functions caused by a drug must interfere with the course of a disease; there thus being two forces acting on the same tissues or organs.

The great epoch of modern physiological investigation was inaugurated by Bichat, and his discoveries were of vast importance. When he came on the stage, therapeutics were in a truly chaotic condition; there being no settled principles whatever which constituted reliable guides for practice. Some, he said, considered materia medica, of all sciences, that which showed to the fullest extent the contradictions of the human mind; but, for his part, he did not think it a science at all. "It is said," he continued, "that the practice of medicine is repulsive. I go farther. I hold that it is impracticable in the present sta'e of our therepeutics." When Bichat, the founder of modern physiology, died, in 1802, Magendie was passing through his course of studies. Thoroughly imbued with the teachings of Bichat, he pursued investigations of similar character with great enthusiasm and success, and was the first to engage in a scientific physiological and therepeutical research. The subject of this was upon poisoning, and such an experimental investigation was only possible after the functions of the spinal nerves had been determined. He next pursued a brilliant series of experiments in regard to the new alkaloid, strychnia, and the result of this was the discovery of the first physiological antagonism, that between strychnia and paralysis. Many have supposed that the first use of this agent in the treatment of paralysis was entirely empirical; but this was not the case. It was prescribed with a definite and scientific view after Magendie had pointed out its exact physiological action, and the result proved how correct were the deductions concerning its therepeutic value which he based upon this.

Having now advanced thus far, let us take up some special instances of physiological antagonism. As early as 1570 Pena and De Lobel published an account of the counteraction of the effect of belladonna berries by means of theriaca, a preparation of opium. After, theriaca was reported as a powerful antidote for poisons of all sorts; but later experience proved that it was for belladonna. By the beginning of the nineteenth century sufficient facts had accumulated to demonstrate the indisputable antagonism between belladonna and opium, and since then the evidence has been still more abundant and conclusive. In the year 1810 Joseph Lipp, and in 1855 Dr. Thomas Anderson, showed how the toxic effects of belladonna were overcome by the use of opium; the dilation of the pupils which had been produced by the former at once disappearing. In July, 1859, Benjamin Bell, of Edinburgh, published a case of poisoning by atropia successfully treated with opium. In January, 1862, Dr. C. C. Lee published in Philadelphia the report of two cases, illustrating the antagonistic effects of the two drugs. The first was one of opium poisoning, and the second one of belladonna poisoning, In his paper Dr. Lee gave a *resume* of the literature of the subject, and also entered with some detail into the history of the adverse experience of Brown–Sequard. Then Dr. William F. Norris, of Philadelphia, gave a complete tabulated sketch of every case, which he could possibly collect, that illustrated the antagonism between the drugs. In 1865 an admirable paper on the subject was published by Mitchell and Moorhouse, of Philadelphia, and since then the cases and papers had greatly multiplied.

The history of this subject is not complete, however, without some reference to the opinions and experiments of those opposing the view of such an antagonism. These authorities, it is well to know, have based their conclusions principally on experiments made upon animals. The most noted of them is Brown-Sequard, and he experimented upon guinea-pigs. Bois used cats for the purpose. Thus, to a cat he gave a dose of morphia almost but not quite sufficient to destroy life, and when the animal had entirely recovered from the effects of this, he gave it a dose of atropia just short of producing death. Then when a sufficient time had elapsed to ensure complete recovery from that, he gave the cat the same doses of morphia and atropia together, and they proved fatal. Onsum, on the other hand, made his experiments on frogs; but whatever are the animals employed, or whatever the methods resorted to, the results were uniformily unfavorable to the existence of an antagonism between the two drugs. None cf these experiments, however, were free from certain sources of error. Harley collected the largest number of facts, but his fundamental error was that he was willing to accept only those cases in which the antagonism extended throughout the whole range of effects.

Although the antagonism between opium and belladona was the first to be discovered, these drugs do not by any means afford the only example of such physiological antagonism. There are, in fact, more perfect antagonisms than this, and such an instance is that between strychnia and disease. Magendie's experiments upon animals with this agent enabled him to suggest what its therapeutic effects would be, and Fouquier applied the suggestions successfully in practical medicine. Magendie said in advance that medicine would derive great benefit from an agent that was capable of producing certain special effects upon the spinal cord, and his opinion was afterward abundantly confirmed by the results of clinical experience. Could any fact more strikingly exhibit the benefit to be derived from physiological experiment?

Later followed the remarkable investigations of Claude Bernard, and the discovery of the physiological effects of woorara. This agent is essentially a paralyzer, and hence it is recommended in cases where spasm is to be overcome. Especially is it indicated in tetanus and hydrophobia, and experience with it thus far seems to show that it is the single remedy which has appeared to have a curative effect in the latter disease. Such discoveries ought certainly to have enough weight to silence forever the silly cavils of anti-vivisectionists.

Next we have the discovery of the antagonism between atropia and physostigma. In 1867 a single experiment was made by Bournesville in proof of this. He first gave a dose of calabar bean to a guinea-pig, and then followed it with a sufficient dose of atropia to counteract its effects. During that year I was making experiments in the same direction with a view to offering their results to the profession, in a paper to be presented in competition for the prize of the American Medical Association. This paper was published in 1869, and in it I distinctly asserted the existence of the antagonism. In the following year the remarkable paper of Thomas R. Frazer, of Edinburgh, appeared on the subject. In the meanwhile, Professor Preyer had published, in 1868, the first part of an elaborate treatise on prussic acid. In the second part, which appeared the following year, he contended that hydrocyanic acid and atropia were physiologically opposed to each other. That this was the case, however, was denied by a number of German authorities, and also by Hare and Keen, of Philadelphia, as well as by myself. In a second paper more recently published, Professor Preyer pays his respects to us all, and proves to his own satisfaction, at least, that we are mistaken.

In 1869 Oscar Liebreich gave us chloral, and demonstrated its antagonism against strychnia. Noted results, in proof of this, were also obtained by the committee appointed by the British Medical Association, with J. Hughes Bennett at its head. The next year Dr. Fothergill, under the auspices of the British Medical Association, showed the antagonism between aconite and digitalis, and in 1877 Huseman's paper on the antagonism of chloral appeared. To such importance, then, has this subject attained, that no study of a medicinal agent is complete until its antagonisms have been determined. As we have seen, the doctrine of contraries was, for a very long period, the leading principle in the field of therapeutics, and it is evident that, even when this prevailed, many instances of antagonism must have been discovered by clinical experience.

(The lecturer then went on to show that the doctrine of physiological antagonisms found its counterpart in the mechanism of the functions of the brain and nervous system. Thus, in the brain, there were centres for the inhibition of reflex action, and reflex action was interfered with when coincident opposing impressions arrived from different points. In this way singultus might be checked by the application of a strong faradic current. In the medulla oblongata there was a centre of extreme sensibility, and above it a spasm centre; and if there were not this check upon the latter the most violent effects would be produced. Hence, the abnormal readiness to react when the iufluence of the cerebrum was withdrawn. In the same way the vascular tonus in the heart was maintained at the normal by the action of antagonistic influences.) The manner in which the cardiac action is affected by opposing influences, he continued, is admirably shown by the effect of cold and heat. If we place the heart of a turtle or a frog, freshly taken from the body of the animal, upon a metallic surface, and apply cold to the latter, we shall find that the organ gradually ceases to contract and dilate; but if we then apply heat in the same way, the heart-beats will at once be resumed. Again, if we drop upon the freshly-exposed heart of a frog a small quantity of serum, contaning muscarin, the organ at once ceases to act, and apparent death is produced; but if we then drop upon the heart a little serum, containing two-tenths per cent. of atropia, it will begin to act again.

The reciprocity of action is a safeguard, and thus danger to the circulation is prevented by a quickening of the pulsations of the heart. A similar mechanism is found in the function of respiration, where the movements of inspiration and expiration are antagonistic, as well as in the control of the sphincters and other physiological phenomena,

We find also that the same principle obtains in the world of physics. I need hardly remind such an audience as this, that if two waves of light, of equal altitude, arriving from opposite directions, meet at half the distance of their respective elevations and depressions, so that the crest of one corresponds to the hollow of the other, both are obliterated, and the result is darkess; nor that if two equal waves of sound meet under similar circumstances, the result is silence. In the same way, when two bodies of equal weight and momentum come together, both are arrested at the point of impact. So, in electricity, when the positive and negative elements are equal, an exact equilibrium is maintained; and in chemistry, too, the great principle is the same. But why multiply examples, when nothing can possibly be more evident than that antagonisms exist everywhere?

I will now ask your attention more particularly to the physiological antagonism between opium and belladonna, and in what follows I shall use the terms opium and morphia and belladonna and atropia synonymously. In the investigation of any such supposed antagonism two inquiries present themselves: *first*, Does the antagonism exist? and *second*, What is its nature? Dr. Norris, of Philadelphia, in the paper before alluded to, presented to the profession the first complete tabulated statement of all the instances of the antagonistic action between the two drugs that had been recorded in medicine up to the date of its publication. I have now added to this list all the cases that have appeared since that time, as well as two others, occurring previously, which seem to have escaped the attention of Dr. Norris. One of these was published in 1854, by Lindsay, and the other in 1855, by Dr. Sibson. The total number now on record is 120. Of these only fifteen cases proved fatal, which is a percentage of 12.5 failures, a result which certainly indicates something more than a *post hoc*. The antagonistic agents which were employed must undoubtedly have averted death; but at the same time it is also necessary to estimate the part that may have been played by other agents, such as emetics, the stomach pump, coffee, flagellation, etc.

The history of the fatal cases is very instructive; for why should the antagonism fail? A number of elements must be taken into consideration in answering this question, and these can perhaps best be brought out by taking a rapid glance at the fifteen cases mentioned.

Case I.—This was reported by Ludwig Pollak in the *Wiener Presse.* The patient was sixty years of age, and four years before had had an apoplectic seizure. He took 36-100 of a gramme, or about five grains, of atropia, with suicidal intent. The report states that a syringeful of morphia (strength not stated) was injected hypodermically, and that no apparent effect being produced by this, another dose, of half the quantity, was employed in the same way.

*Commentary.*—The condition of the brain in this case was undoubtedly very unfavorable for a recovery; but especially is the failure to be attributed to the inefficient method in which the morphia was administered. If we assume that the solution was of the usual strength and the syringe of the usual size, the amount could not have exceeded three grains altogether. The proper method to have pursued here would have been to inject one-quarter or one half a grain of morphia every twenty minutes, until some pronounced effect had been produced upon the respiration and the pupils. The patient having had a cerebral lesion, however, there would of course have been less chance of a favorable result than if this had not been the case.

Case II.—Reported by James Seaton in the *Medical Times and Gazette*, in 1859. This was one of ten cases of poisoning by belladonna berries, the patient being a child. Eight minims of tincture of opium were given every half hour at first, and afterward the dose was increased to twelve minims. Death ensued at the expiration of twenty-nine hours from the time the poison was taken. Altogether, seventy-nine minims of laudanum were taken—which is about equal to three grains of crude opium—and this quantity was entirely too little to counteract the effect of a lethal dose of belladonna. The post-mortem showed, however, that the heart was flabby and that there was pleural effusion and adhesion.

Case III.—Reported by Dr. Samuel W. Gross in *The American Journal of the Medical Sciences* for October, 1869. The patient was a lady, forty-three years of age, and she took four pills containing three grains of atropia, which were sent her through the mistake of a druggist. Among the other symptoms there was complete muscular relaxation, with the exception that there was trismus of the lower jaw. At first an enema and quantities of emetics were given, and then half a grain of morphia was administered hypodermically, after which the respirations became twenty to the minute. The effect being temporary, however, the dose was repeated, when the respirations again became twenty. The faradic current was also employed, and respiration was kept up artificially. Later, a third half-grain of morphia was given, but the respirations now becoming fourteen to the minute, and stertorous in character, it was decided not to give any more opium. Artificial respiration was continued, and the use of the faradic current and flagellation after a time seemed to restore the patient to partial consciousness. Subsequently she appeared to sink into a natural sleep, the respirations then being eighteen. Just as the medical men were about to leave her, however, the toxic symptoms returned. Veratrum ointment was now rubbed vigorously into the skin along the spine, and artificial respiration being resumed, she seemed to improve again. About this time some of the urine was injected into a cat, when it was found that the animal's pupils became dilated. No more morphine was given, and the patient finally expired. At the autopsy it was found that the superficial veins were congested and that the veins of the brain were also very full, while there was general softening of the cerebral tissue. The tissue of the heart was also soft and easily torn; so that this case can hardly be regarded as a failure for the doctrine of antagonisms. In view of the considerable quantity of atropia taken, moreover, scarcely less than six grains of morphia were needed to counteract its effect. After the first dose of one-half grain, the trismus of the jaw ceased and the respiration became twenty. A little later no inconsiderable portion of the belladonna poison must have been already eliminated, since, as we have seen, the injection of the patient's urine into a cat was sufficient to produce one of the most marked physiological effects of the drug. It is of no little importance in such cases to estimate the quantity of the poison that is being eliminated. The case was a very unfavorable one, however, from the condition of affairs revealed by the autopsy, and there can be little doubt that the heart was in an advanced stage of fatty degeneration.

Case IV.—Reported by Dr. Southam in the *British Medical Journal*, in 1868. The patient was thirty-eight years of age, and took two ounces of laudanum. Nearly eighteen hours had elapsed before he came under treatment, and by that time the pupils were contracted to a pin's point, and the respirations had become reduced to four in the minute. The hypodermic injection of 1-36 of a grain of atropia was given, with the effect of raising the respirations to nearly the normal number; but this beneficial effect was very brief in duration. Accordingly, the dose was repeated, until five in all had been taken. At the autopsy there was found to be marked congestion of the lungs.

*Commentary.*—The poison had already been absorbed when the treatment was undertaken, and there was an obvious deficiency in the employment of the atropia. As the whole of the opium remained in the blood, the whole of the atropia ought to have been given at once instead of in divided doses, so as to make a more profound and prompt impression. In a similar case Milner Fothergill administered one grain of atropia at once, and with entire success. Of course the long duration of the toxic symptoms before the remedy was given constituted an unfavorable element in the case.

Case V.—Reported by Dr. Paget in the *British Medicvl Journal.* A child three and a half years old swallowed an unknown quantity of laudanum, and in order to counteract its effects, 1-100 of a grain of sulphate of atropia was given in four hours after the taking of the poison. No appreciable result following this, a second dose of half the quantity was promptly administered, and after this it was noticed that the pupils dilated slowly, while the extremities grew cold and the respiration began to fail. Artificial respiration was then resorted to, and, in addition, coffee was given and ambulation and flagellation employed. There was then a temporary improvement, but death took place in twenty-eight hours.

*Commentary.*—The important effect of atropia on the pupil and the brain is well shown in this case. Here it is probable that the first injection of the remedy was sufficient, for after the second the coldness of the extremities was noticed. It would have been decidedly better to wait and see what the effect of the first dose was before resorting to a second. It seems altogether likely, therefore, that here the amount of opium taken was not very large, and that the too free use of the antagonistic agent, together with the other active treatment employed, proved too much for the strength of a child of three and a half years.

Cases VI. and VII.—Reported by Dr. Haynes, of Philadelphia. One of the patients took an ounce and a half of laudanum, and the other one ounce of crude opium. In both these cases the doses of atropia were much too small, and hence the source of failure was sufficiently plain.

Cases VIII. to XIII.—These were reported by James Johnson, and occurred in the Chinese hospital at Shanghai. In all there was profound opium-poisoning, and in each but one dose of atropia was administered, the amount given being totally inadequate to counteract the effects of the poison.

Case XIV.—Reported by Horatio C. Wood, of Philadelphia. In this case one and a half grains, and afterwards one and a quarter grains of opium, had been given for the relief of cholera morbus. To antagonize the profound narcotic effect produced by it, fourteen hypodermic injections of atropia were administered, the whole amount given aggregating one-quarter of a grain. Here the underlying disease, no doubt, had much to do with the fatal result, and such a case is of small value, owing to the uncertainty which is thus introduced into any estimation of the antagonistic action cf the two agents.

Case XV.—Reported by Dr. Beddoe. The patient took a teaspoonful of belladonna liniment, and death ensued in sixteen hours. Three injections of morphia of one grain each were given in all. It was found that decided stupor followed the second dose, and hence the third should have been withheld, for after that the patient breathed stertoriously, notwithstanding the fact that the pupils remained widely dilated.

It was noted by the observers present that toward the last the case precisely resembled one of opium-poisoning, with the exception that the pupils were dilated instead of contracted; and this leads me to say that the condition of the pupils in such cases cannot always be depended upon as an index of the actual effect of the antagonistic agents upon each other and upon the system. But, owing to the lateness of the hour, I shall have to defer until next week the further consideration of this subject.

